# Mechanosorptive creep of Norway spruce on the tissue scale perpendicular to grain

**DOI:** 10.1515/hf-2025-0115

**Published:** 2026-02-18

**Authors:** Alessia Ferrara, Falk K. Wittel

**Affiliations:** ETH Zurich, Institute for Building Materials, HIF E 28, Laura-Hezner-Weg 7, 8093 Zürich, Switzerland

**Keywords:** wood tissues, mechanosorptive creep, transverse directions, orthotropy

## Abstract

Mechanosorptive creep strain (MCS) is a strain component that can dominate the overall deformation behavior of wood components under load and changing moisture. In this work, the MCS behavior of Norway spruce (*Picea abies*) tissues is investigated in the anatomical directions perpendicular to grain and different loading degrees (LD). The MCS is evaluated through a strain decomposition of the total strain, which is determined using a computer-controlled digital image correlation (DIC) system. It is shown that the common assumption of a scalar relation between mechanosorptive creep compliance (MCC) and the orthotropic, moisture-dependent elastic one is questioned by experiments.

## 1 Introduction

The behavior of wood under both varying mechanical load and moisture content (*mc* or *ω*) is very rich. Unfortunately, this complexity is only weakly considered by constitutive models, primarily due to a sparse experimental basis with differing interpretations. However, it would be important, when thinking about the recent boost in timber engineering, where cross-laminated timber (CLT) and glue-laminated timber (GLT) products are used to build skyscrapers (Villamizar Santamaría 2024) or for self-shaping CLT that relies on moisture changes (Grönquist et al. 2019). The material symmetry of a growing tree results in an orthotropy with radial (*R*), tangential (*T*), and longitudinal (*L*) anatomic directions. Typically, one distinguishes different deformation components like elastic, plastic, hygro-expansion, viscoelastic creep (VCS), and mechanosorptive creep strain (MCS) that add up to the total strain (Fortino et al. 2009; Hassani et al. 2015; Mackenzie-Helnwein and Hanhijärvi 2003; Reichel and Kaliske 2015a). Note that combinations of VCS and MCS (Angst and Malo 2013) or viscoplastic and plastic strain (Reichel and Kaliske 2015b) were also proposed; however, these are ultimately different interpretations of the same experimental basis. This work focuses on the MCS that is often neglected in timber engineering, even though bending experiments have shown that the deflection due to MCS can be up to 20 times larger than that under constant climate (Grossman 1976). MCS is the creep strain that evolves due to moisture changes under load, which explains why an isolated determination is not possible. MCS is virtually time-independent and influenced by the magnitude of moisture change below fiber saturation and the moisture level, rather than by the moisture flux. Its origin within the hierarchical material is still not fully resolved, but observed on all scales, resulting in inter- and intra-scale interactions. The cellular tissue scale bridges tracheids and the macroscopic behavior, and thus deserves special attention that has not been given to MCS on the tissue scale in different anatomical directions in the past.

Mechanosorptive creep in wood was first described in the late 1950s and early 1960s by Armstrong and Christensen (1961) and Armstrong and Kingston (1960). However, similar behavior was already observed in concrete (Pickett 1942) and later reported for other hygroscopic materials such as wool (Mackay and Downes 1959; Nordon 1962), paper (Byrd 1972a,b), and also in synthetic fibres (Wang et al. 1993, 1990). An overview of mechanosorptive creep across different materials is provided by Alfthan (2004). From the onset, researchers speculated on its relationship to the existence and change in hydrogen bond density associated with moisture changes. Reviews on MCS were published by Grossman (1976), Hoffmeyer and Davidson (1989), Holzer et al. (2007), Mårtensson (1994), and Schniewind (1968). The latest review includes atomistic and micro-mechanical perspectives (Navi and Stanzl-Tschegg 2009). In the past, several inconsistent theories have been proposed for the origin of MCS. Boyd (1982) interpreted deformations in MCS tests on the molecular level with molecular kinetics theory and with a lenticular trellis model that can qualitatively explain observed processes. Navi et al. (2002) proposed that the MCS is generated by slips between polymer chains that are cross-linked by hydrogen bonds. In this spirit, Hoffmeyer and Davidson (1989) proposed a slip-plane theory for localized slip planes forming in the *S*_2_ layer. However, MCS is already observed at stress levels below the threshold for slip-plane formation and also under tension, where no slip-planes have been observed so far. In addition, it does not explain the difference between moisture-dependent VCS and MCS. Mukudai and Yata (1986) explained MCS on the cell wall ultra-structure, where, due to differential shrinkage of layers, relative slip between (*ML* + *P* + *S*_1_) and (*S*_2_ + *S*_3_) layers could be triggered. Unfortunately, their validation is questionable due to possible preparation artefacts. Entwistle (2005) relates MCS to shear stress components along the cellulose-matrix interface and points to similarities between the mechanism generating the MCS and the ones responsible for the large plastic strains in wet compression wood loaded in tension, namely, breaking and reforming of hydrogen bonds between parallel polymer chains. Amando de Barros and Wittel (2024) took up several of these ideas and proposed a fiber bundle model (FBM) with moisture and history-dependent slip-stick behavior where elastic, swelling, plastic, VCS, and MCS emerge naturally from a single slip-stick mechanism.

Several interesting observations surround MCS, including larger creep rates for desorption than for absorption, the emergence of extremely large shear strains beyond typical failure limits, and the progressive reduction of MCS increments with increasing cycle number. Hanhijirvi and Hunt (1998) discovered an influence of the MCS on the VCS rate and reported on a limit for MCS (Hunt 1980, 1989, 1999; Hunt and Shelton 1988). However, the existence of an MCS limit has not been confirmed so far (Mohager and Toratti 1992). At constant moisture, strains are frozen, but can be partially recovered after unloading through moisture cycles, with greater recovery during absorption. This phenomenon is also referred to as the moisture-induced shape memory effect, characterized by both recoverable and irrecoverable strains. In the FBM approach (Amando de Barros and Wittel 2024), this behavior emerges when slip-back events have lower thresholds than advancing slips.

Most MCS studies are made under bending to put the material in the *L*-direction simultaneously under tension and compression (Bengtsson 2001; Houška 1997; Koc and Houška 2002; Mårtensson 1994; Rémond et al. 2013; Zhou et al. 1999). Inhomogeneous, evolving stress fields, strong moisture gradients due to transport, and deviating creep behavior under compression and tension complicate the interpretation of such experiments to an extreme extent, resulting in multiple misinterpretations of MCS in the past. However, some studies on purely tensile or compressive macroscopic samples, also in transverse directions, were made (Huč and Svensson 2018; Pérez-Pena et al. 2016; Ranta-Maunus 1993; Svensson and Toratti 2002; Toratti and Svensson 2000; Wu and Milota 2007), highlighting the importance of direct measurements in different anatomic directions. Unfortunately, many of these works are on different species, do not report on decomposed strains, or do not comprise a sufficient number of moisture cycles.

This study investigates the mechanosorptive creep behavior of Norway spruce (*Picea abies*) on tissue samples to minimize the effect of moisture-gradient-induced creep due to residual stresses. It is focused on the two transverse anatomical directions (radial and tangential), where MCS is most pronounced, across various loading degrees (LD). A fully automated test rack with a computer-controlled digital image correlation (DIC) system and five parallel axes, located in a glove box with moisture control, ensures identical hygric and mechanical loading conditions across different samples (Ferrara and Wittel 2025a). The mechanosorptive creep strain is extracted from the total strain via strain decomposition, employing a novel incremental scheme that not only mitigates the effects of inaccurate mapping of moisture content as a function of relative humidity *ω*(*RH*), since samples may not reach equilibrium under variable humidity, but also reduces uncertainty related to the elastic and hygroexpansion parameters. Then the mechanosorptive creep compliance (MCC) is modeled by a series of three Kelvin–Voigt (KV) type elements in response to moisture changes, rather than in the time domain (Hassani et al. 2015). The experiments show that the scalar relation between MCC and the orthotropic, moisture-dependent elastic compliance tensor, which is typically assumed at the macroscopic scale (Fortino et al. 2009), does not hold at the tissue scale.

## 2 Materials and methods

Mechanosorptive creep in wood is among the most challenging phenomena to characterize experimentally. Its complexity arises from the intricate coupling between hygric and mechanical deformations, requiring highly controlled and sensitive measurement setups and advanced evaluation procedures for strain decomposition. First, the preparation of samples for tensile creep testing (Section 2.1) is described, along with the experimental setup. Since only relative humidity can be controlled in the climate chamber, the quantification highly depends on the accompanying results of the dynamic vapor sorption (DVS) measurements described in Section 2.2. This is fundamental, as moisture content is the control parameter for the evaluation procedure, namely the strain decomposition procedure for isolating the mechanosorptive strain from the total measured strain. Section 2.3 first describes the validation of the procedure on calculated clean data, and then its application to the real data of transverse strains.

### 2.1 Material selection and experimental setup

**Figure 1:**
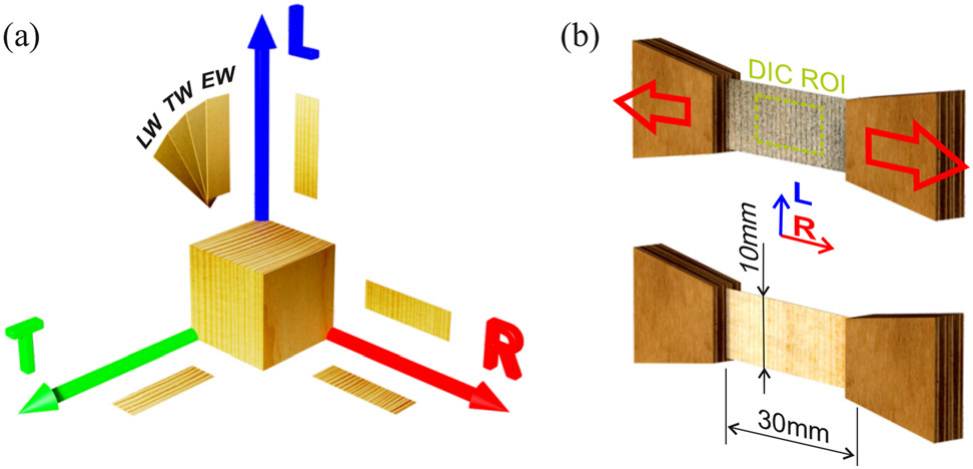
Spruce tissues for all combinations of directions {*LR*, *RL*, *RT*, *TR*, *LT*} (a), and exemplary *RL* samples prepared with glued-on load application pieces from plywood, and speckled for DIC measurements via an airbrush (b).

All samples were obtained from the same Norway spruce (*Picea abies*) log used in earlier studies by the authors (Ferrara and Wittel 2024, 2025a). After conditioning from the green state to 65 % RH/20 °C, small blocks ((20 × 15 × 50) mm^3^) were cut from sapwood for slicing tissues in different anatomical orientations. The sample preparation followed the procedure described in Ferrara and Wittel (2025a) to obtain samples in the transverse directions, namely *RL*, *RT*, *TR* (see Figure 1), where the first letter denotes the loading direction and the second the transverse width, which contains multiple growth rings in different orientations.

**Figure 2:**
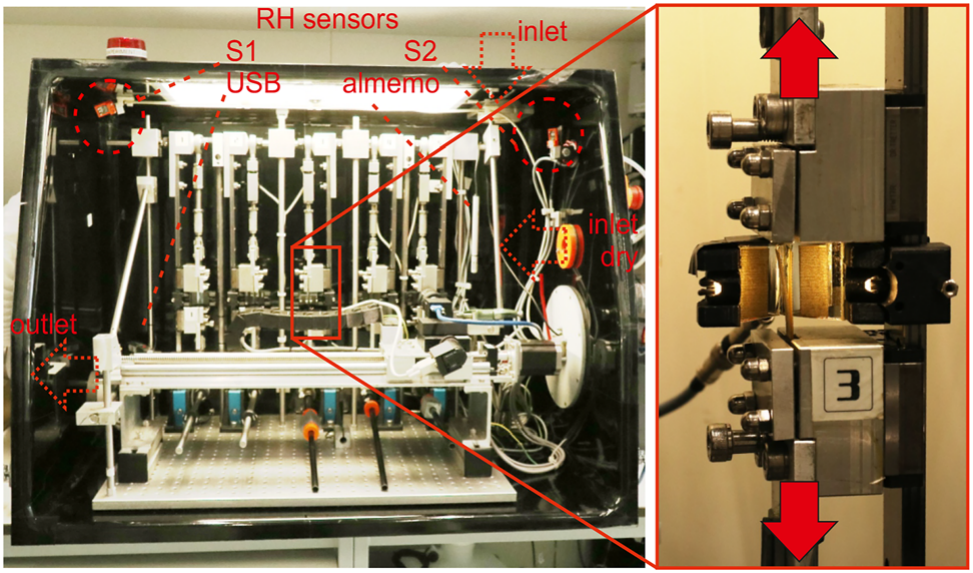
Custom-designed test rack for tensile creep testing on thin tissue samples, equipped with five sample holders (inset) to allow simultaneous testing of multiple samples. The humidity sensors (S1, S2), along with the inlet and outlet of the ventilation system, are marked in red to highlight their positions. In the inset, red arrows indicate the directions of the forces acting on the sample.

The automated 5-axis creep-rack, mounted inside a climatized glove box (see Figure 2a), allows testing of multiple samples held on low-friction linear guides and loaded via lever bars with static weights, while a dual-mirror system combined with a two-axis camera captures both surfaces for accurate DIC measurements. This setup was previously described in detail in Ferrara and Wittel (2025a) and also the sample geometry remains similar: 50 mm in length and 10 mm in width with thicknesses of about 0.35 mm–0.40 mm. A double-slice configuration, gluing the ends, was applied to all samples to increase the thickness. Samples were glued with a fixture to trapezoidal loading pieces of 2 mm-thick plywood at their ends to ensure a smooth load introduction and sample alignment (see sample holder in Figure 2b) and finally speckled with fine black and white acrylic paint to obtain the characteristic pattern for strain measurement via DIC.

An overview of the samples that entered this study is provided in Table 1. Note that at least *n* = 4 for each case were valid, while experiments exhibiting early failure, evident pre-damage from sample preparation or clamping, or unreliable DIC measurements were omitted.

**Table 1:** Sample sizes for the respective tissue types, grouped by loading degree (LD).

Loading direction	Transverse direction	LD [%]		
		30	40	50
*R*	*L*	5		4
*R*	*T*	4		5
*T*	*R*		4	

The samples were tested under changing humidity in a cyclic pattern between 30 % and 90 % RH at a period Δ*T* of about 2 h (see Section 2.2). Furthermore, two loading levels (LDs) corresponding to approximately 30 and 50 % of the tensile strength $\sigma_{ii}^{f}$ measured at 90 % RH (Ferrara and Wittel 2024) were applied to {*RL*, *RT*}. For *TR*, however, only the 40 % LD yielded valid results. Higher loads caused the samples to fail right after the first moisture cycle, likely due to excessive stress concentrations near the clamps from hygroexpansion. Lower LDs, e.g. 30 %, could not be tested reliably under these varying conditions as they approach the minimum load capacity of the testing rack. Once samples were loaded at 30 % RH, humidity was cycled between 30 % and 90 % RH, ramping up/down at ±10 % RH steps every 4 min with 38 min hold periods at each extreme. This sequence was repeated 8–9 times under load, after which the load was released for 2 relaxation cycles.

**Figure 3:**
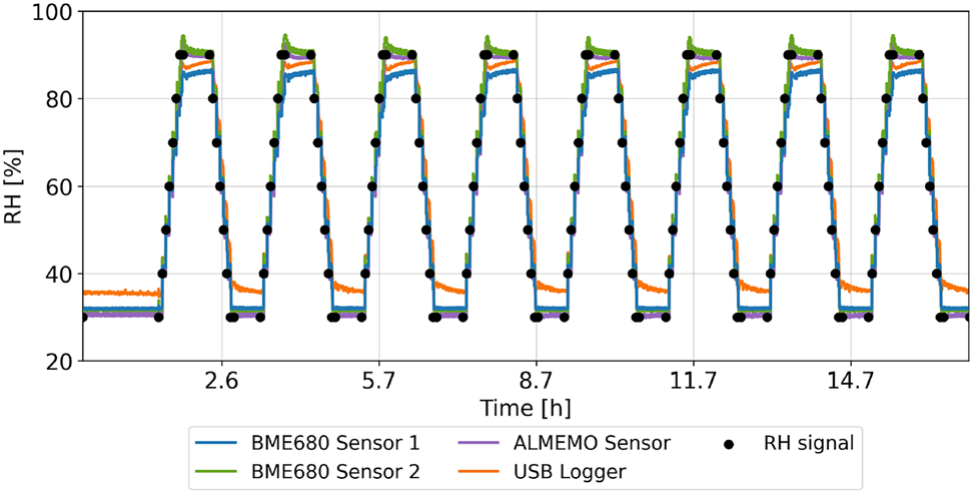
Time evolution of relative humidity (RH) inside the glove box measured by the two BME680 sensors (blue and green), the ALMEMO sensor (violet), and the USB data logger (orange). The black markers indicate the signals sent to the control system to set the target RH.

The relative humidity inside the glove box is controlled by a Python script on a Raspberry Pi 3B+, which uses readings from two moisture sensors (Bosch BME680, ±3 % RH accuracy) located at opposite sides of the chamber (see Figure 2a) to mantain target humidity within ±2 % RH. Dry or moist air streams are introduced as needed, while a ventilation system ensures uniform conditions. The control system’s long-term stability and dynamic response were verified by replicating the mechanosorptive moisture-cycling protocol and comparing readings from additional sensors (ALMEMO Ahlborn FH A646-E1 and USB data logger EL-USB-2) at various positions. Figure 3 presents an example of RH data recorded for a 2 h-hold at 30 % RH followed by 8 full cycles. The measurements from all sensors agree within a negligible discrepancy, demonstrating both the system’s rapid response to humidity changes and the ventilation system’s ability to maintain rather uniform conditions in the box.

### 2.2 Sorptivity of spruce tissues

**Figure 4:**
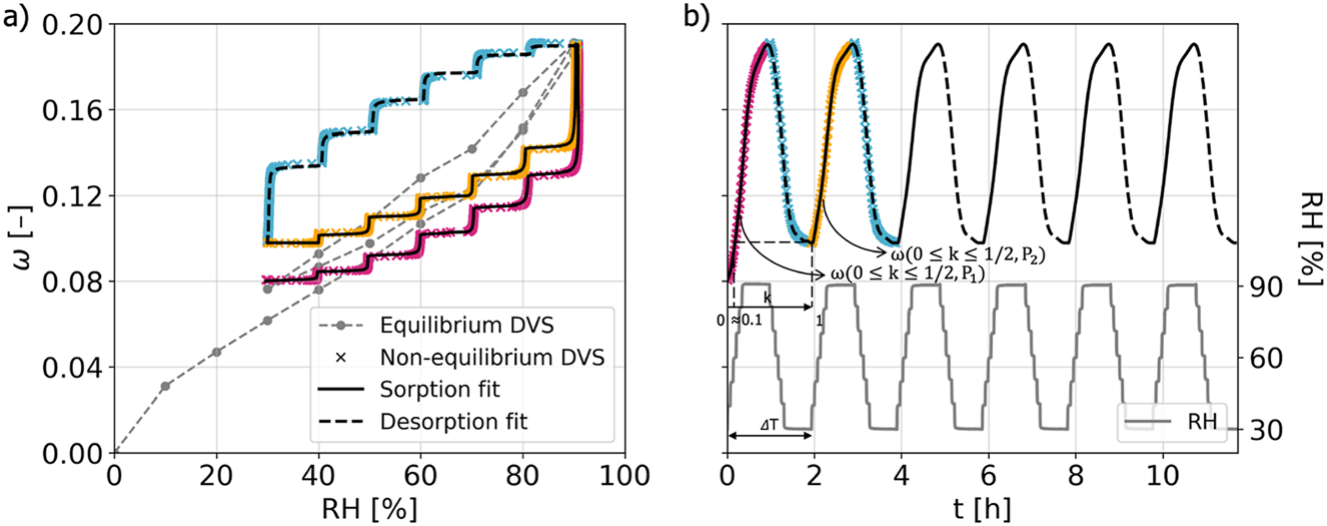
DVS test results on a *RL* tissue sample. (a) Moisture content (*ω)* as a function of relative humidity (RH): equilibrium DVS (grey dashed line) and non-equilibrium DVS (cross markers: magenta = first sorption *ω*(*k*, *P*_1_), with 0 ≤ *k* < 1/2, orange = second sorption *ω*(*k*, *P*_2_), light blue = desorption), with cubic spline fits for sorption (black solid line) and desorption (black dashed line). (b) Example of a periodic cubic-spline fit representing *ω* as a continuous function of time and RH history (grey solid line).

The evaluation of mechanosorptive tests requires the accurate mapping of moisture content *ω* with RH (or time), since under varying humidity conditions, the samples may not be fully equilibrated to each humidity step. For this purpose, tailored DVS measurements were conducted on a *RL* tissue sample (approximately 8 mg) that followed the predefined RH profile of the test, using an automated sorption balance (DVS Advantage ET85, Surface Measurement Systems Ltd.). Note that the tests were performed under stress-free conditions. Although sorption behavior may depend on the stress state, its effect on moisture content is likely negligible in the context of mechanosorptive creep. The results of the DVS measurements (staircase-shaped datasets) are overlaid in Figure 4a for direct comparison. After equilibrating at 30 % RH, moisture uptake closely follows the equilibrium curve in the initial sorption phase, indicating that the sample remains near equilibrium. However, as RH rises, the actual moisture content progressively deviates from the sorption curve, only reconverging by the end of the 90 % RH hold phase. During desorption, water removal proceeds more slowly due to the moisture-dependent diffusion coefficient (Skaar 1988). As a result, it fails to return to its equilibrium state by the end of the 30 % RH hold. In the subsequent sorption phase, moisture uptake follows a similar staircase pattern, reconverging to the same point by the end of the 90 % RH hold, repeating thereafter. Thus, the first sorption phase differs, while the desorption curves remain consistent. Note that starting from a moist state would not eliminate this history-dependent discrepancy, as the first turning point would still not correspond to the initial equilibrium moisture content. For comparison, the equilibrium sorption isotherm (grey dashed line in Figure 4a) between 30 % and 90 % was measured, following the procedure outlined in Koch et al. (2024) (sample predried at 60 °C in N_2_ (N5.0); partial pressure *P*/*P*_0_ steps 0–0.98 at 25 °C; equilibrium d*m*/d*t* < 0.0005 %/min; N_2_ flow 200 sccm).

Since the actual evolution of *mc* during experimental humidity cycling diverges from the equilibrium isotherms, the non-equilibrium measurements are taken as reference for its mapping, but with the initial half-cycle being different from all subsequent ones. Moreover, because fitting *ω* as a function of time (periodic cubic spline) is more straightforward than fitting it against RH (staircase-shaped curve), the time-based spline is adopted for post-processing so all the strain components (see Section 2.3) are expressed as a function of time *t* and *ω*. Figure 4b illustrates an example of this periodic time-based spline fit of *ω*. Since these measurements are representative for spruce as the tested slice contains multiple growth rings, they were then applied to all sample types.

### 2.3 Incremental decomposition scheme for mechanosorptive strain calculation

The evaluation of the experimental data is based on two time series, one for each speckled surface with lens distortion correction applied. Surface strain fields are computed at each time *t* by DIC of the reference and current images using the Ncorr MATLAB package (Blaber et al. 2015) (estimated strain accuracy of about ±3 ⋅ 10^−5^). A scalar strain value in the loading direction is calculated by averaging the strain field. The two surface scalars are further averaged to yield a single scalar tensile strain *ɛ*^tot^(*t*), as in Ferrara and Wittel (2025a). Finally, *ɛ*^tot^(*t*) is synchronized with moisture *ω*(*t*) and the corresponding stress *σ*, calculated from the initial cross section and the applied load.

The total strain *ɛ*^tot^ has to be decomposed into its hygroexpansion *ɛ*^*ω*^, elastic *ɛ*^el^, viscoelastic *ɛ*^ve^, and mechanosorptive *ɛ*^ms^ component, which are all strongly moisture-dependent, namely:

(1) $$\varepsilon^{\text{tot}}=\varepsilon^{\omega }+\varepsilon^{\text{el}}+\varepsilon^{\text{ve}}+\varepsilon^{\text{ms}}.$$

Note that plastic strain components are not considered separately and, if any, are comprised in the MCS. Assuming that hygroexpansion, elasticity, and viscoelasticity are already well captured by existing constitutive models, mechanosorption remains the component to be identified. By rearranging Eq. (1), one can obtain *ɛ*^ms^ = *ɛ*^tot^ − *ɛ*^*ω*^ − *ɛ*^el^ − *ɛ*^ve^, but, in practice, *ɛ*^*ω*^, *ɛ*^el^, and *ɛ*^ve^ all depend on the moisture content. Therefore, any error in *ω* propagates into *ɛ*^ms^. To reduce it, *ω* was accurately mapped using the experimental humidity profile (see Section 2.2), and a novel incremental decomposition procedure for calculating MCS is proposed and validated.

Building on the precise moisture mapping, the incremental scheme is founded on the observation that corresponding points in each humidity cycle occur under identical humidity conditions and, therefore, share the same moisture content, so they will have the same hygro-elastic strain. By summing the strain increases between corresponding points in consecutive humidity cycles, one can extract the VCS and MCS. To split off the VCS, its evolution must be calculated under varying moisture and load conditions.

To validate the procedure, the measured moisture history *ω*(*t*) and a stress history *σ*(*t*) are applied to compute all strain components as functions of *ω*(*t*) using prescribed material parameters (from macroscale measurements for hygroexpansion, *RL* tissue samples for elastic and viscoelastic components, and arbitrarily chosen values for mechanosorptive one) and superimpose them to obtain a total calculated strain ɛ*^tot^ (Eq. (1)). In accordance with the analytical model of Hassani et al. (2015), the individual strain components are computed for any direction. They are summarized in scalar form, as only uniaxial situations are regarded:

*ɛ*^*ω*^: The hygroexpansion strain is proportional to increments of moisture, as in
(2) $$\varepsilon^{\omega }\left(\omega \right)=\alpha \left[\omega \left(t\right)-\omega_{0}\right],$$
  where *ω*_0_ is the initial reference moisture content and *α* is the hygro-expansion coefficient. *α* is assumed to remain constant and independent of variations in moisture. The values *α*_*R*_ = 0.182; *α*_*T*_ = 0.343 in [%/%] are obtained from macroscale measurements (samples containing multiple growth rings, approximately (4 × 30 × 110) mm^3^) of the same tree as in this study.
*ɛ*^el^: The elastic strain is calculated from Hooke’s law as
(3) $$\varepsilon^{\text{el}}\left(\omega \right)=C_{0}^{-1}\left(\omega \left(t\right)\right)\cdot \sigma \left(t\right),$$
  where the moisture-dependent elastic compliance $C_{0}^{-1}\left(\omega \left(t\right)\right)$ is determined by a quadratic fit of the mean tensile elastic measurements (Ferrara and Wittel 2024), and then evaluated at *ω*(*t*).
*ɛ*^ve^: The VCS is expressed by a series of *N* = 4 KV-elements, following previous studies (Ferrara and Wittel 2025a,b), in the time domain:
(4) $$\varepsilon^{\text{ve}}\left(t\right)=\overset{N}{\underset{i=1}{\sum }}\varepsilon_{i}^{\text{ve}}\left(t\right)=\sigma \overset{N}{\underset{i=1}{\sum }}C_{i}^{-1}\left(1-{\text{e}}^{-t/\tau_{i}}\right),$$
  where $\varepsilon_{i}^{\text{ve}}$ , $C_{i}^{-1}$ , and *τ*_*i*_ are, respectively, the strain, compliance, and characteristic time of the *i*^th^-element. While the characteristic times were fixed *a priori* to *τ*_*i*_ = [0.1, 1, 10, 100] in h, each moisture-dependent $C_{i}^{-1}$ is obtained by a quadratic fit of the available mean data in Ferrara and Wittel (2025a), as for elastic compliances, and then evaluated at *ω*(*t*). Since both stress and compliance vary with time, each element is governed by the rate equation
(5) $${\dot{\varepsilon }}_{i}^{\text{ve}}+\frac{1}{\tau_{i}}\varepsilon_{i}^{\text{ve}}=\frac{1}{\tau_{i}}C_{i}^{-1}\sigma \left(t\right),$$
  that gives for each finite time increment Δ*t* = *t*_*n*+1_ − *t*_*n*_
(6) $$\varepsilon_{i,n+1}^{\text{ve}}=\varepsilon_{i,n}^{\text{ve}}+\frac{C_{i}^{-1}\sigma \left(t\right)-\varepsilon_{i,n}^{\text{ve}}}{\tau_{i}}\Delta t.$$
*ɛ*^ms^: The MCS is formulated analogously to the VCS above, but in the moisture domain (Fortino et al. 2009; Hanhijärvi and Mackenzie-Helnwein 2003; Hassani et al. 2015). It is modeled as a series of *M* = 3 KV-elements with fixed characteristic moistures *μ*_*j*_ = [1, 10, 100] in [%] (consistent with Hassani et al. (2015)) and compliance tensors $C_{j}^{-1}$ :
(7) $$\varepsilon^{\text{ms}}\left(\omega \right)=\overset{M}{\underset{j=1}{\sum }}\varepsilon_{j}^{\text{ms}}\left(\omega \right)=\sigma \left(t\right)\overset{M}{\underset{j=1}{\sum }}C_{j}^{-1}\left(1-{\text{e}}^{\omega /\mu_{j}}\right),$$
  where $C_{j}^{-1}=\left[1,6,8\right]$ in GPa^−1^ are arbitrarily chosen for the one-dimensional verification calculations. Note that for the experimental data, the $C_{j}^{-1}$ are obtained by least-squares fits of the extracted *ɛ*^ms^. Because both stress and compliance vary with moisture, each element is governed by the rate equation
(8) $${\dot{\varepsilon }}_{j}^{\text{ms}}+\frac{\left|\dot{\omega }\right|}{\mu_{j}}\varepsilon_{j}^{\text{ms}}=\frac{\left|\dot{\omega }\right|}{\mu_{j}}C_{j}^{-1}\sigma \left(t\right),$$
  that gives for each finite moisture increment $\left|\Delta \omega \right|=\left|\omega_{n+1}-\omega_{n}\right|$ corresponding to Δ*t* = *t*_*n*+1_ − *t*_*n*_
(9) $$\varepsilon_{j,n+1}^{\text{ms}}=\varepsilon_{j,n}^{\text{ms}}+\frac{\left|\Delta \omega \right|}{\Delta t}\frac{C_{j}^{-1}\sigma \left(t\right)-\varepsilon_{j,n}^{\text{ms}}}{\mu_{j}}\Delta t.$$

**Figure 5:**
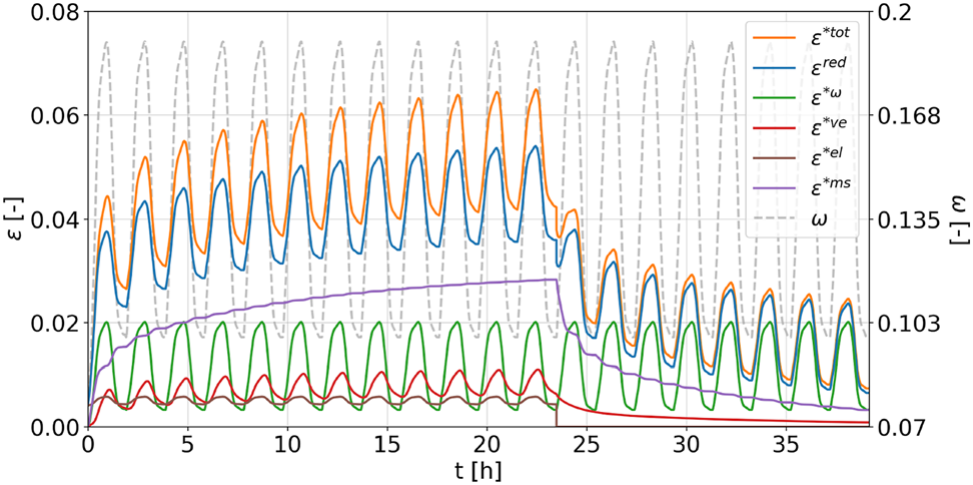
Example of the evolution of hygroexpansion ɛ*^ω^, elastic ɛ*^el^, viscoelastic ɛ*^ve^, mechanosorptive ɛ*^ms^ calculated using Eqs. (2)–(9) (Hassani et al. 2015), and the resulting total strain ɛ*^tot^ over *N*_*P*_ = 20 moisture cycles (12 under tensile load and 8 unloaded) for the purpose of validating the incremental decomposition procedure. The reduced strain *ɛ*^red^ is calculated by subtracting the viscoelastic strain from the total strain and represents the contribution of all non-viscoelastic strain components.

**Figure 6:**
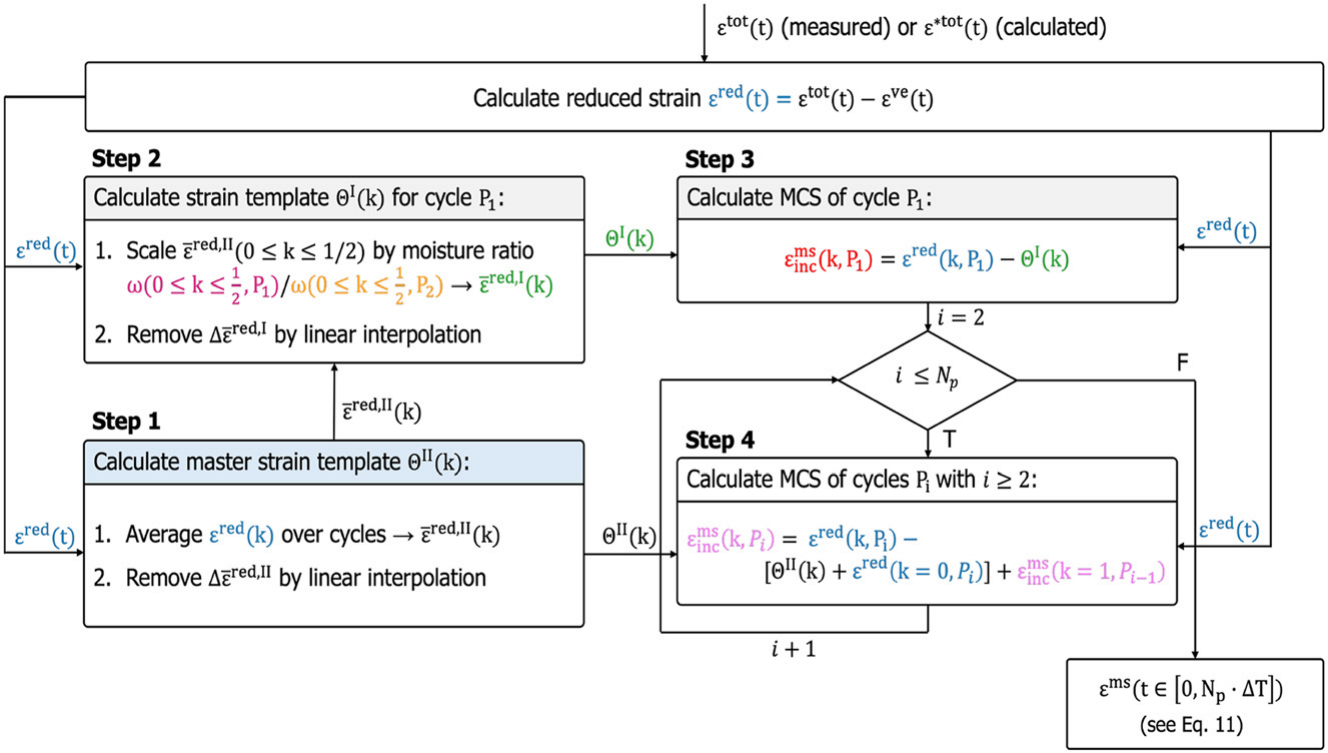
Workflow of the incremental scheme for mechanosorptive creep strain (MCS) calculation. Colors correspond to line segments and cycles in Figures 4, 7, and 8. Note that the calculated values, marked by a star, are not separately shown.

For validating the strain decomposition procedure, each strain component (hygroexpansion ɛ*^ω^, elastic ɛ*^el^, viscoelastic ɛ*^ve^, mechanosorptive ɛ*^ms^) is computed over *N*_*P*_ = 20 moisture cycles, with 12 under tensile load and 8 unloaded to allow for relaxation, as shown in Figure 5. The number of cycles is chosen arbitrarily and could be any for validation purposes. Starting from the total strain ɛ*^tot^(*t*), calculated by summing the individual strain components, the incremental decomposition procedure illustrated in Figure 6 is applied to extract the MCS $\varepsilon_{\text{inc}}^{\text{ms}}$ . This is then compared with the calculated MCS ɛ*^ms^ used to compute ɛ*^tot^(*t*) to verify whether the curves align. If they do, the incremental decomposition procedure is considered successful in isolating the MCS contribution.

As each cycle *P*_*i*_, except the first half-cycle *P*_1_ with *k* ∈ [0, 1/2), undergoes an identical moisture evolution (see Figure 4a) at the same relative cycle time *k*, the sample is at the same moisture state for each value of *k* ∈ [0, 1] that corresponds the period Δ*T*. Therefore, respective strain differences at identical relative times *k* eliminate the hygro-elastic strain components *ɛ*^*ω*+el^(*k*). What cannot be eliminated is the history-dependent viscoelastic creep strain *ɛ*^ve^ that evolves during the hygro-mechanical loading. One has to calculate its evolution using Eqs. (4)–(6) with the moisture-dependent constitutive parameters obtained beforehand (Ferrara and Wittel 2025a). Using the calculated VCS evolution, one can define a reduced strain *ɛ*^red^ = *ɛ*^tot^ − *ɛ*^ve^ for simplicity. *ɛ*^red^ is still composed of the hygro-elastic strain *ɛ*^*ω*+el^, which can be eliminated by the cyclic differences, the MCS, and possible plastic strains that remain a part of the MCS.

**Figure 7:**
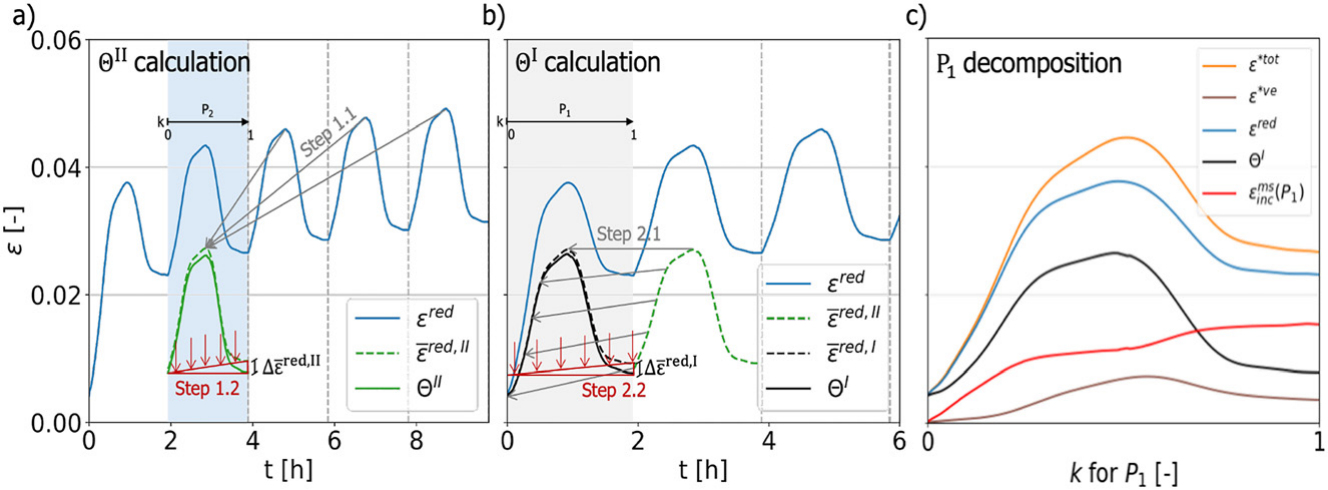
Incremental decomposition scheme for the calculation of mechanosorptive strain during moisture cycles *P*_*i*_ with steps explained in Figure 6. (a) Calculation of strain templates Θ^II^(*k*) and (b) Θ^I^(*k*). (c) Decomposition of cycle *P*_1_ in relative cycle times *k*.

The MCS incremental decomposition procedure only works from cycle *P*_2_ onwards, determining the cycle increments, while the MCS of cycle *P*_1_ needs to be determined separately. The procedure is based on building templates Θ from the reduced strain *ɛ*^red^ that describe the repeating hygro-elastic strain *ɛ*^*ω*+el^(*k*) = *ɛ*^*ω*^(*k*) + *ɛ*^el^(*k*) per cycle. Hence, two templates are needed, one for cycle *P*_1_ that is called Θ^I^ and one for all the other cycles called template Θ^II^:

Θ^II^: To obtain a smooth template for the hygro-elastic strain *ɛ*^*ω*+el^, one can average over all cycles *P*_*i*_ with *i* ≥ 2 at identical stress *σ*. However, the cycles are more alike at later stages, as the cyclic mechanosorptive strain increase decelerates, giving a cleaner *ɛ*^*ω*+el^(*k*). Therefore, only the last few cycles under load enter the calculation. First, one collapses the cycles by plotting them as a function of the relative cycle time *k* and shifting them so peak values of *ɛ*^red^(*k*) overlay. As they are quite similar and to reduce noise, an averaged ${\bar{\varepsilon }}^{\text{red}}\left(k\right)$ is calculated as shown in Figure 7a (Step 1.1 in Figure 6). This way, there is still a relative increase in MCS for the averaged ${\bar{\varepsilon }}^{\text{red,II}}\left(k\right)$ (since ${\bar{\varepsilon }}^{\omega +\text{el}}\left(k=0\right)={\bar{\varepsilon }}^{\omega +\text{el}}\left(k=1\right)$ ) that corresponds to the averaged MCS increment $\Delta {\bar{\varepsilon }}^{\text{red,II}}={\bar{\varepsilon }}^{\text{red,II}}\left(k=1\right)-{\bar{\varepsilon }}^{\text{red,II}}\left(k=0\right)$ . To eleminate MCS from ${\bar{\varepsilon }}^{\text{red,II}}\left(k\right)$ a linear increase over the cycle up to $\Delta {\bar{\varepsilon }}^{\text{red,II}}$ is assumed and subtracted from ${\bar{\varepsilon }}^{\text{red,II}}\left(k\right)$ , as shown in Figure 7b (Step 1.2 in Figure 6). The final template Θ^II^ is now only the mean hygroelastic strain ${\bar{\varepsilon }}^{\omega +\text{el}}\left(k\right)$ and can be applied for the decomposition of all cycles *P*_*i*_ with *i* ≥ 2. Note that a new template is required for load changes, since the elastic strain *ɛ*^el^ differs.
Θ^I^: Unfortunately, Θ^II^(*k*) cannot be directly applied to cycle *P*_1_, since *ω*(*k*) differs for cycle *P*_1_ due to the initial sorption phase that starts at the equilibrium moisture content. It is corrected by scaling the averaged strain ${\bar{\varepsilon }}^{\text{red, II}}\left(0\geq k\geq 1/2\right)$ , previously determined from the calculations of template Θ^II^ (Step 1.1) by the moisture ratio *ω*(0 ≥ *k* ≥ 1/2, *P*_1_)/*ω*(0 ≥ *k* ≥ 1/2, *P*_2_) between the sorption phases of cycle *P*_1_ and *P*_2_, respectively, as shown in Figure 7b (Step 2.1 in Figure 6). Note that this superposition approach assumes linear correlations for *ɛ*^*ω*^, *ɛ*^el^ with moisture *ω* within this 2 % moisture deviation. In the next step, the MCS increment of the first cycle $\Delta {\bar{\varepsilon }}^{\text{red,I}}$ needs to be subtracted, which only makes sense for identical moisture states. Therefore, one cannot take the value at *k* = 0, but must take the one that corresponds to the first sorption phase, where the moisture is *ω*(*k* = 1), as reference (*k* ≈ 0.1). Once $\Delta {\bar{\varepsilon }}^{\text{red,I}}$ is determined, its linear increase over the entire cycle is subtracted from ${\bar{\varepsilon }}^{\text{red,I}}$ , as shown in Figure 7b (see Step 2.2 in Figure 6) to obtain the strain template Θ^I^(*k*) for cycle *P*_1_. Note that possible non-linear behavior will result in small deviations during the cycle. However, values at every end of the cycle should be correct, except for a negligible discrepancy due to the linear nature of the correction procedure for the templates.

With the two templates Θ^I^, Θ^II^, one can now calculate the mechanosorptive strain for each cycle (Steps 3 and 4 in Figure 6) following


(10) $$\varepsilon_{\text{inc}}^{\text{ms}}\left(k,P_{i}\right)=\left\{\begin{array}{cc} \varepsilon^{\text{red}}\left(k,P_{1}\right)-\Theta^{I}\left(k\right) & \;\text{if }i=1\\ \varepsilon^{\text{red}}\left(k,P_{i}\right)-\left[\Theta^{II}\left(k\right)+\varepsilon^{\text{red}}\left(k=1,P_{i-1}\right)\right]+\varepsilon^{\text{ms}}\left(k=1,P_{i-1}\right) & \;\text{if }i\geq 2. \end{array}\right.$$

The overall MCS *ɛ*^ms^(*t*) is simply the consecutive sequence of all $\varepsilon_{\text{inc}}^{\text{ms}}\left(k,P_{i}\right)$ for cycles *i* = 1, …, *N*_*P*_ with the period Δ*T* that can be written with the indicator function **1**_[*a*,*b*)_(*t*) that is defined as **1**_[*a*,*b*)_(*t*) = 1 if *a* ≤ *t* < *b*, and **1**_[*a*,*b*)_(*t*) = 0 otherwise:

(11) $$\varepsilon_{\text{inc}}^{\text{ms}}\left(t\right)=\overset{N}{\underset{i=1}{\sum }}\varepsilon^{\text{ms}}\left(\frac{t-\left(i-1\right)\Delta T}{\Delta T},P_{i}\right)\cdot 1_{\left[\left(i-1\right)\Delta T,\;i\Delta T\;\right)}\left(t\right).$$

Note that for additional cycles at a different load, a new template, e.g. Θ^III^, needs to be determined and those cycles need to be appended. This was made for the recovery part with no load, but not explicitly shown for compactness, as the procedure is identical.

**Figure 8:**
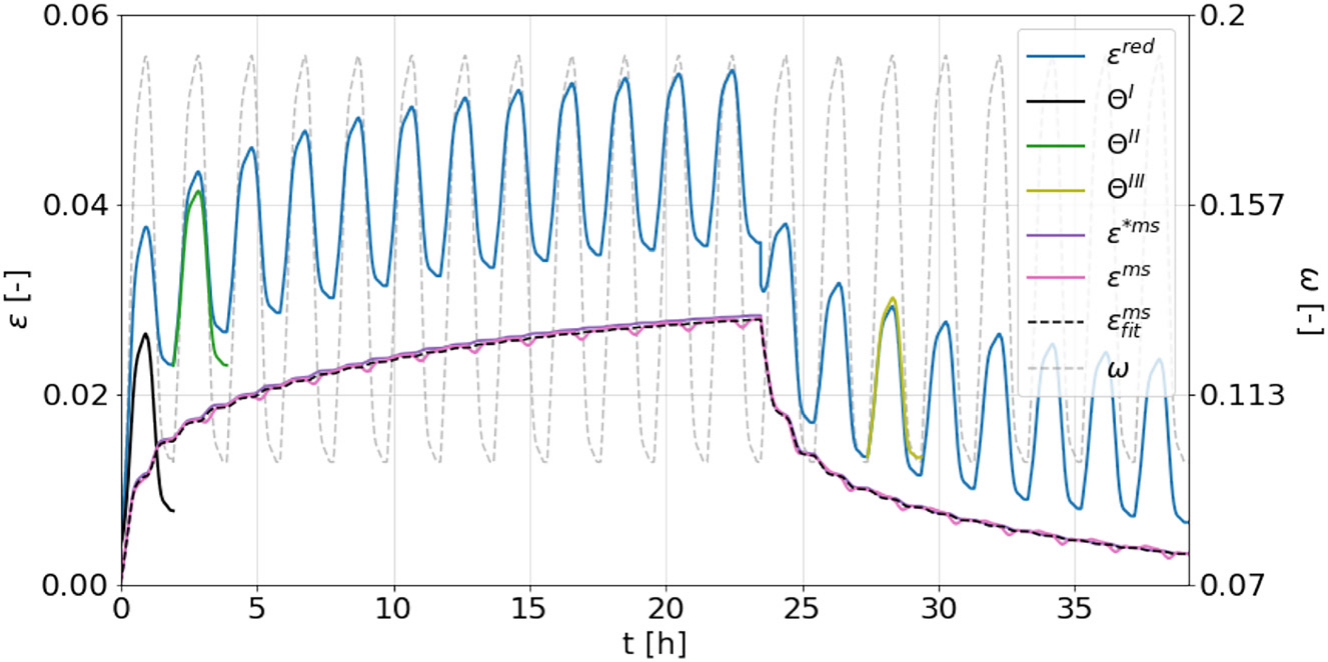
Comparison of calculated mechanosorptive strain ɛ*^ms^(*t*) with the extracted one *ɛ*^ms^(*t*) and its Kelvin–Voigt model fit $\varepsilon_{\text{fit}}^{\text{ms}}\left(t\right)$ for moisture cycles under load and recovery, as well as strain templates Θ^I^–Θ^III^.

Now that the decomposition procedure is complete, the resulting mechanosorptive creep strain *ɛ*^ms^(*t*) is compared in Figure 8 with the initially calculated MCS ɛ*^ms^(*t*), which was part of ɛ*^tot^(*t*). The curves align remarkably well, confirming the validity of the incremental decomposition scheme. This approach also remains valid during unloading and, more generally, for any change in load throughout the experiment, provided that a new template is generated. The resulting MCS can then be fitted with the mechanosorptive KV-model (see Eq. (7)) to determine the optimized parameters $C_{j}^{-1}=\left[0.8,6.1,7.9\right]$ GPa^−1^, which are also in close agreement with those used to calculate ɛ*^ms^(*t*) ( $C_{j}^{-1}=\left[1,6,8\right]$ GPa^−1^). Note that the scheme remains valid regardless of the specific model, equation, or arbitrarily generated data series used to calculate the initial MCS ɛ*^ms^(*t*), as the key requirement is simply to have a prescribed curve of reasonable magnitude contributing to the total strain, which allows verification that the decomposition scheme can successfully isolate it.

The validated decomposition procedure can now be applied to real and noisy experimental data *ɛ*^tot^(*t*) from DIC, synchronized with the corresponding *σ*(*t*) and *ω*(*t*) recordings. Finally, *ɛ*^ms^(*t*) is modeled using Eq. (7), consistently employing the characteristic moistures *μ*_*j*_ = [1, 10, 100] %, while the unknown parameters $C_{j}^{-1}$ are determined via least-squares fitting analogous to the verification example. Finally, average MCS are computed across all samples for each investigated situation from the individual KV-series, and a mean KV-series ${\bar{\varepsilon }}^{\text{ms}}\left(t\right)$ is obtained by fitting the average curve. The MCS are converted into compliance *M*_*c*_(*t*) by dividing the strain by the applied stress and compared with the elastic compliances.

## 3 Results and discussion

**Figure 9:**
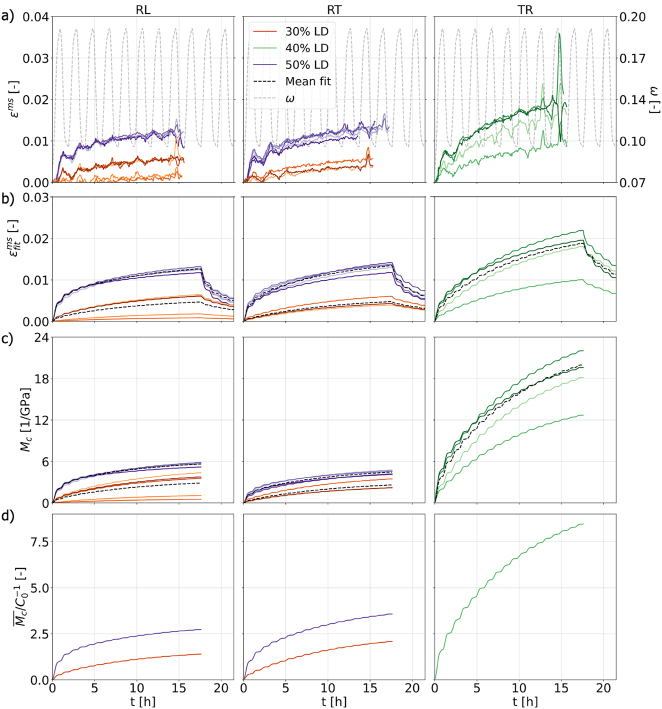
Evolution of mechanosorptive strain and compliance under cyclic humidity and different loading degrees LD (30 and 50 % for {*RL*, *RT*}, 40 % for *TR*). (a) Mechanosorptive strains *ɛ*^ms^ from incremental decomposition scheme (full strains dataset available at DOI: 10.17632/rsrsw8h7mv.1). (b) Fitted strains $\varepsilon_{\text{fit}}^{\text{ms}}$ from Kelvin–Voigt model. (c) Mechanosorptive compliances *M*_*c*_ in GPa^−1^. (d) Normalized compliances $\overset{®}{M_{c}}/C_{0}^{-1}$ using $C_{0}^{-1}$ from Ferrara and Wittel (2024) at 0.08 moisture content.

With this experimental setup, it can be investigated how moisture changes under mechanical load influence the creep behavior of different spruce tissues in the transverse directions {*RL*, *RT*, *TR*}, where the MCS is most pronounced, and explore the dependence on the loading degree LD. Figure 9 provides an overview of all results. First, the isolated mechanosorptive strains *ɛ*^ms^(*t*) through successive moisture cycles are shown for all samples of each investigated situation (Figure 9a). These curves were obtained by applying the incremental decomposition procedure from Section 2.3 to the total strains *ɛ*^tot^(*t*) measured by DIC for each sample. Note that one *RT* sample under 30 % LD and all unloading phases yielded unreliable data and were therefore excluded from the final evaluation. Although the incremental procedure is valid for any load change, two unloading cycles proved insufficient to resolve MCS increments during unloading with an acceptable accuracy.

The MCS were fitted to the three-element KV-model described in Section 2.3. Figure 9b shows the strains computed via Eq. (7) using the fitted compliances $C_{j}^{-1}$ , alongside the mean fitted response for each combination of tissue type and LD. Note that one can calculate the average MCS response for the moisture history of this study at arbitrary times using the KV-element compliances from Table 2 and Eq. (7). This fitting procedure filters noise in the raw data and gives the possibility to extend the dataset to the relaxation phase, where load is removed and MCS decreases. It is evident that in the *R*-direction, higher applied loads result in increased creep deformation. At first glance, the MCS of *RL* and *RT* appear similar for each LD, but *RL* samples are actually subjected to lower absolute load due to their lower strength compared to *RT* (Ferrara and Wittel 2024). When considering the tangential orientation, *TR* exhibits higher strain at 40 % LD than the radial tissues at 50 % LD.

**Table 2:** Element compliances $C_{j}^{-1}$ in GPa^−1^ of Kelvin–Voigt series fits of the average creep response for varying loading degrees LD (30 and 50 % for {*RL*, *RT*}, 40 % for *TR*) under moisture cycling between 30 % and 90 % RH. For each case, the mean coefficient of determination *R*^2^ represents the average of the *R*^2^ values from the fits to the individual experiments.

Loading direction	Transverse direction	LD	$C_{j}^{-1}$ [GPa^−1^]	Mean *R*^2^
*R*	*L*	30 %	[1.3 ⋅ 10^−3^, 3.7 ⋅ 10^−1^, 3.1]	0.7
*R*	*L*	50 %	[2.6 ⋅ 10^−3^, 2.4, 3.9]	0.9
*R*	*T*	30 %	[2.2 ⋅ 10^−2^, 2.4 ⋅ 10^−3^, 2.5]	0.6
*R*	*T*	50 %	[1.2 ⋅ 10^−4^, 1.6, 3.8]	0.9
*T*	*R*	40 %	[1.4 ⋅ 10^−1^, 2.2, 20.2]	0.9

The difference between anatomical directions is even more expressed for the mechanosorptive compliance (MCC) curves *M*_*c*_(*t*) (see Figure 9c), which were calculated from the ratio of the fitted strains and the applied stress during the loading phase. *M*_*c*_ in the *R*-direction remains multiple times lower than the one in the tangential direction, mirroring trends reported for both elastic and viscoelastic deformations (Ferrara and Wittel 2024, 2025a) as creep under constant or changing moisture involves micro-structural adjustments of the cellular structure similar to elastic deformation. In the transverse direction, deformation is mainly governed by cell wall bending inducing stress localization, while radial loading also induces stretching through undulating load paths, enhancing performance and making the *R*-direction stiffer than *T* (Gibson and Ashby 1997). Moreover, microstructural features such as rays strongly affect compliance in thin spruce samples: under radial loading they stretch and contract, under tangential loading they open acting like microstructural voids. Interestingly, *RL* exhibits greater compliance than *RT*, despite sharing the same loading axis, because slicing exposes cut-open cells and rays across the surface of *RL*, unlike in *RT* where they appear only at the narrow edges (Ferrara and Wittel 2025a). The MCC curves for different load levels become closer but do not fully collapse, as in the case of viscoelastic creep compliances (Ferrara and Wittel 2025a), indicating that the mechanosorptive response does not follow a simple linear scaling regime.

Finally, to show how MCC scales with the elastic compliances $C_{0}^{-1}$ reported in previous work (Ferrara and Wittel 2024), Figure 9d gives the ratio $\bar{M_{c}}\left(t\right)/C_{0}^{-1}$ , where $\bar{M_{c}}\left(t\right)$ is the average of the fitted MCC and a constant value of $C_{0}^{-1}\left(\omega =0.08\right)$ . The rise of the normalized MCC underscores that moisture history affects mechanosorptive but not elastic compliance. Moreover, the relevance of the moisture history depends on the orientation, with the T-direction exhibiting a surprisingly large amplification up to a factor of 9 in this loading sequence. Clearly, additional moisture cycles under load would result in even higher values. In the resulting data, it cannot be therefore observed any limit for MCS, especially in T-direction, as reported by Hunt (1980, 1989, 1999). This could be due to a high LD or the uniaxial stress case that could be realized. Note that normalization by compliance values at other moisture states or even following the moisture evolution only changes the picture in a negligible way.

**Figure 10:**
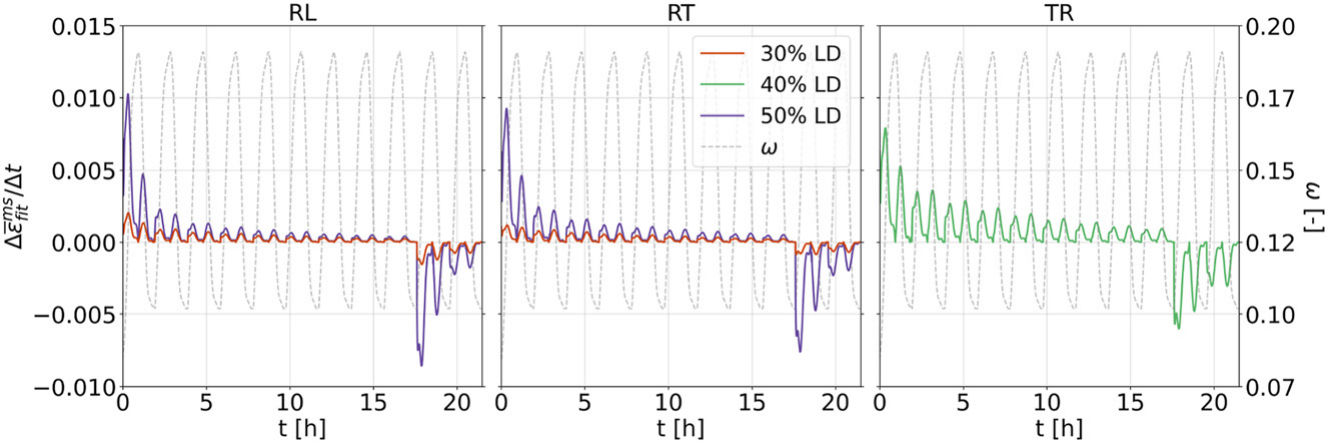
Evolution of mean fitted mechanosorptive strain rate $\Delta {\bar{\varepsilon }}_{\text{fit}}^{\text{ms}}/\Delta t$ under cyclic humidity and different loading degrees LD (30 and 50 % for {*RL*, *RT*}, 40 % for *TR*).

Although the MCS continuously increases over successive moisture cycles, its rate exhibits a distinct behavior, characterized by a sharp rise during the first sorption, followed by a continued but gradually decreasing peak rate. Figure 10 illustrates the evolution of the mean fitted MCS rate $\Delta {\bar{\varepsilon }}_{\text{fit}}^{\text{ms}}/\Delta t$ . In each direction, the initial steep rise is evident up to a peak, after which the rate decelerates as the *mc* approaches its maximum. This again highlights the dependence of MCS on moisture change. When *mc* reverses and decreases, the rate rises sharply again before slowing near the minimum *mc* at the end of the lower holding phase. The markedly high MSC rate peak highlights the stronger impact of the initial phase of the first moisture cycle. By contrast, subsequent cycles show much smaller and progressively diminishing peaks, with no significant difference between sorption and desorption. Interestingly, the *T*-direction exhibits a less pronounced decrease in rate and maintains a higher rate in the later cycles compared to the *R*-direction, despite being at a different LD. Moreover, a clear LD-related pattern emerges: the lower the LD, the lower the rate but also the less steep the decline of the peaks. Note that the MCS increment corresponds to the area underneath the rate curves.

**Figure 11:**
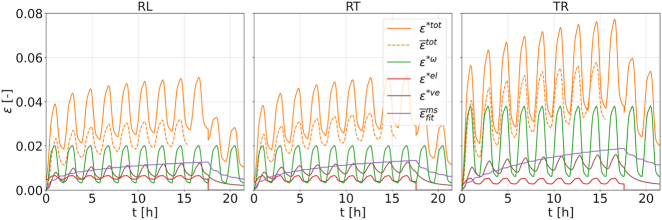
Calculated total strain ɛ*^tot^ as the sum of calculated hygroexpansion ɛ*^ω^, elastic ɛ*^el^, and viscoelastic ɛ*^ve^ strains, and the mean fitted mechanosorptive strain ${\bar{\varepsilon }}_{\text{fit}}^{\text{ms}}$ extracted from the DIC-measured total strain *ɛ*^tot^ using incremental decomposition scheme (mean ${\bar{\varepsilon }}^{\text{tot}}$ ). Results correspond to *RL*, *RT* at 50 % loading degree and *TR* at 40 %.

To relate MCS to the other strain components, one can plot the absolute values of strain for the different directions and their decomposition into the different strain contributions. In Figure 11, the calculated total strain ɛ*^tot^ is obtained as the sum of the mean fitted MCS $\varepsilon_{\text{fit}}^{\text{ms}}$ (at 50 % LD for *RL*, *RT* and 40 % LD for *TR*) and the other calculated strain components (ɛ*^ω^, ɛ*^el^, and ɛ*^ve^) according to Eqs. (2)–(4). For comparison, the mean total strain ${\bar{\varepsilon }}^{\text{tot}}$ obtained from DIC measurements is also provided. It is striking to observe that MCS slightly exceeds the VCS and the elastic one by far. This points to the importance of considering MCS in the transverse directions in a dedicated way in constitutive models of wood for timber engineering.

## 4 Conclusions

This study sheds light on the complex phenomena of creep under changing climatic conditions in spruce. It was possible to obtain continuous strain data for a large number of samples and relatively long testing periods due to the full automation of a climatized test rack. Additionally, multiple loading axes assure identical moisture histories of the samples. The isolation of MCS in wood is non-trivial, as the measured total strain is composed of hygroexpansion, elastic, viscoelastic, and mechanosorptive strain components. The decomposition requires a good knowledge of the viscoelastic behavior of the samples, which was characterized in previous work (Ferrara and Wittel 2025a), as it can only be separated by calculating its evolution, as well as the evolution of moisture. By analytically generating clean data from a rheological model (Hassani et al. 2015), it was developed and validated a novel incremental strain decomposition scheme that reduces the effects of non-equilibrium moisture mapping under variable RH and reduces uncertainty in the hygro-elastic behavior. Accompanying DVS tests using the identical cycling RH profile ensured precise moisture mapping.

The conducted experiments reveal striking directional dependencies in mechanosorptive creep, most notably a dominant compliance in the T-direction that cannot be explained by elastic analogies alone or compliance changes under respective moisture states. In principle, MCC increases with humidity changes, as also demonstrated by the present findings, and is time-independent. However, since the samples are never fully equilibrated (see Figure 4), one cannot expect plateaus. This points to possible size effects that require further investigation. The results show that MCS rates under sorption surpass the ones under desorption only for the first cycle in loading and relaxation. Consecutive cycles do exhibit identical rates without any dominance. Moreover, at the cell-wall level, internal stresses may alter sorptive behavior (Barkas 1949), with volumetric stress locally shifting moisture content: higher in tension and lower in compression. This disrupts the assumption of constant moisture fields within the cell wall and causes tracheid corners acting as hinges to soften and amplify directional effects, which were observed in this study. These effects are further superimposed with residual stresses from delayed moisture equilibration, which can drive creep strains. This clearly disproves the common assumption of a simple scalar relationship between elastic and mechanosorptive compliance tensors and highlights a lack of understanding of creep deformation mechanisms on the cellular scale.

While this work only explored the transverse directions, future work should be extended to the longitudinal direction, as strain variations in the L-direction were beyond the accuracy of the experimental setup. Isolated tissues, namely early, transition, and late wood tissues perpendicular to grain, would also be very interesting; however, the load ranges were below the experimental design. Such investigations could clarify the contribution of each isolated tissue to the cumulative behavior of a growth ring and further advance the understanding of mechanosorptive creep behavior across all anatomical directions and tissue types. With results from accompanying plasticity experiments on the tissue scale, one could further decompose the total strain by subtracting the calculated plastic evolutions from MCS. Finally, an advanced cellular model that incorporates stress-sorption couplings could shed light on the directional dependencies.

## Abbreviations and symbols

DIC: digital image correlation
DVS: dynamic vapor sorption
KV: Kelvin–Voigt
LD: loading degree
*mc*: moisture content
MCC: mechanosorptive creep compliance
MCS: mechanosorptive creep strain
*R*, *T*, *L*: radial, tangential, longitudinal direction
RH: relative humidity
VCC: viscoelastic creep compliance
VCS: viscoelastic creep strain
**1**_[*a*,*b*)_: indicator function
*α*: hygroexpansion coefficient
$C_{0}^{-1}$: elastic compliance
Δ*t*: time increment
Δ*T*: moisture cycle period
*ɛ*^el^ (*ɛ**^el^): elastic strain (calculated using prescribed material parameters)
*ɛ*^ms^ (*ɛ**^ms^): mechanosorptive strain (calculated using prescribed material parameters)
$\varepsilon_{\text{inc}}^{\text{ms}}$: mechanosorptive strain from incremental decomposition procedure
$\varepsilon_{\text{fit}}^{\text{ms}}$ $\left({\bar{\varepsilon }}_{\text{fit}}^{\text{ms}}\right)$: mechanosorptive strain fitted with KV-model (mean)
$\varepsilon_{j}^{\text{ms}}$ , $C_{j}^{-1}$ , *μ*_*j*_: mechanosorptive strain, compliance, characteristic time of *j*th KV-element
*ɛ*^red^ $\left({\bar{\varepsilon }}^{\text{red}}\right)$ , $\Delta {\bar{\varepsilon }}^{\text{red}}$: reduced strain (mean), mean MCS increment
*ɛ*^tot^ (*ɛ**^tot^): total strain (calculated using prescribed material parameters)
*ɛ*^ve^ (*ɛ**^ve^): viscoelastic strain (calculated using prescribed material parameters)
$\varepsilon_{i}^{\text{ve}}$ , $C_{i}^{-1}$ , *τ*_*i*_: viscoelastic strain, compliance, characteristic time of *i*th KV-element
*ɛ*^*ω*^ (*ɛ**^*ω*^): hygroexpansion strain (calculated using prescribed material parameters)
*ɛ*^*ω*+el^ $\left({\bar{\varepsilon }}^{\omega +\text{el}}\right)$: hygro-elastic strain (mean)
*k*: relative normalized times of moisture cycles
*M*: number of KV-elements for MCS computation
*M*_*c*_: mechanosorptive compliance
*N*: number of KV-elements for VCS computation
*P*_*i*_, *N*_*P*_: moisture cycle index, number of moisture cycles
*σ*: stress
*t*: time
Θ^I^, Θ^II^, Θ^III^: strain templates
*ω*: moisture content
$\bar{\omega }$: moisture content rate
*ω*_0_: reference moisture content
